# The effect of bundling medication-assisted treatment for opioid addiction with mHealth: study protocol for a randomized clinical trial

**DOI:** 10.1186/s13063-016-1726-1

**Published:** 2016-12-12

**Authors:** David H. Gustafson, Gina Landucci, Fiona McTavish, Rachel Kornfield, Roberta A. Johnson, Marie-Louise Mares, Ryan P. Westergaard, Andrew Quanbeck, Esra Alagoz, Klaren Pe-Romashko, Chantelle Thomas, Dhavan Shah

**Affiliations:** 1 Center for Health Enhancement Systems Studies, University of Wisconsin-Madison, Madison, WI 53706 USA; 2 School of Journalism and Mass Communication, University of Wisconsin-Madison, Madison, WI 53706 USA; 3 Communication Arts Department, University of Wisconsin-Madison, Madison, WI 53706 USA; 4 Department of Medicine, University of Wisconsin School of Medicine and Public Health, Madison, Wisconsin 53705 USA; 5 Access Community Health Centers, Madison, WI 53715 USA; 6 Mass Communication Research Center, School of Journalism and Mass Communication, University of Wisconsin-Madison, Madison, WI 53706 USA

**Keywords:** Technology, mHealth, Addiction, Medication-assisted treatment, Opioids, Smartphone, HIV, HCV

## Abstract

**Background:**

Opioid dependence has devastating and increasingly widespread consequences and costs, and the most common outcome of treatment is early relapse. People who inject opioids are also at disproportionate risk for contracting the human immunodeficiency virus (HIV) and hepatitis C virus (HCV). This study tests an approach that has been shown to improve recovery rates: medication along with other supportive services (medication-assisted treatment, or MAT) against MAT combined with a smartphone innovation called A-CHESS (MAT + A-CHESS).

**Methods/design:**

This unblinded study will randomly assign 440 patients to receive MAT + A-CHESS or MAT alone. Eligible patients will meet criteria for having an opioid use disorder of at least moderate severity and will be taking methadone, injectable naltrexone, or buprenorphine. Patients with A-CHESS will have smartphones for 16 months; all patients will be followed for 24 months. The primary outcome is the difference between patients in the two arms in percentage of days using illicit opioids during the 24-month intervention. Secondary outcomes are differences between patients receiving MAT + A-CHESS versus MAT in other substance use, quality of life, retention in treatment, health service use, and, related to HIV and HCV, screening and testing rates, medication adherence, risk behaviors, and links to care. We will also examine mediators and moderators of the effects of MAT + A-CHESS.

We will measure variables at baseline and months 4, 8, 12, 16, 20, and 24. At each point, patients will respond to a 20- to 30-min phone survey; urine screens will be collected at baseline and up to twice a month thereafter. We will use mixed-effects to evaluate the primary and secondary outcomes, with baseline scores functioning as covariates, treatment condition as a between-subject factor, and the outcomes reflecting scores for a given assessment at the six time points. Separate analyses will be conducted for each outcome.

**Discussion:**

A-CHESS has been shown to improve recovery for people with alcohol dependence. It offers an adaptive and extensive menu of services and can attend to patients nearly as constantly as addiction does. This suggests the possibility of increasing both the effectiveness of, and access to, treatment for opioid dependence.

**Trial registration:**

ClinicalTrials.gov, NCT02712034. Registered on 14 March 2016.

**Electronic supplementary material:**

The online version of this article (doi:10.1186/s13063-016-1726-1) contains supplementary material, which is available to authorized users.

## Background

Opioid dependence has devastating consequences for patients, family members, and communities. In 2012, an estimated 2.1 million Americans had opioid use disorders (OUDs) related to prescription opioids, and 467,000 had OUDs related to heroin [1]. The total volume of opioids prescribed in the health care system has risen steeply in recent years. In 1991, about 76 million prescriptions were written for opioids; in 2013, about 207 million prescriptions were written [2]. A growing proportion of people with OUDs started their use of opioids by taking prescription opioids. Emergency department visits related to the nonmedical use of opioids rose from 144,600 in 2004 to 305,900 in 2007 [3] and unintentional overdose deaths from opioids have more than quadrupled since 1999, reaching their highest level ever in 2014 [2, 4]. OUDs also have been a primary driver of the increased spread of human immunodeficiency virus (HIV) and hepatitis C virus (HCV) in many rural and suburban communities in the US [5, 6].

Existing treatments for OUDs often fail. Following detoxification from opioid dependence, early relapse is the most common outcome [7]. After inpatient treatment, the vast majority of patients relapse within a year, often within the first few months [8]. Medication-assisted treatment (MAT) arose when methadone became available in the 1960s. Along with other supportive services, such as peer support, MAT has been shown to increase rates of recovery from OUD [9]. Yet those who receive MAT still do not generally maintain long-term abstinence [8, 10].

Access to treatment is an enormous challenge, with only 10.7% of OUD patients who needed treatment in 2012 receiving it [11]. Effective treatment is complex and demanding because OUDs are chronic diseases that require ongoing medication, behavioral counseling, and overdose protection, as well as screening and treatment for infectious disease and comorbid psychiatric disease [2]. Effective treatment is also complex because affected populations differ in the etiology and course of their addiction, motivation for treatment, and reasons for relapse, including notable differences between men and women [12]. For example, women tend to progress more quickly from the start of substance use to the start of dependence, have a higher rate of cooccurring mood and anxiety disorders, and have better outcomes on buprenorphine than on methadone [12]. Finally, retention in treatment, which is known to reduce drug use [13], remains a challenge in treating OUDs [14–19]—so much so that treatment retention is often itself regarded as a desired outcome [20].

Testing and links to care for HIV and HCV are essential for people who inject opioids. Those who inject drugs are at greater risk of contracting HIV and are less successfully linked to [21–23] and retained in [24–26] clinical care. Antiretroviral therapy is recommended for all patients living with HIV, but treatment is under-used [27] and often suboptimally effective [23, 28] among people who inject drugs. HCV occurs primarily in people who inject drugs, with 90% of older injection-drug users infected [29–31]. HCV is the most common blood-borne infection in the US [32] and the most common cause of end-stage liver disease and the need for liver transplants.

The randomized clinical trial described here assesses the extent to which the considerable challenges of effectively treating OUDs can be addressed by an mHealth intervention. Specifically, we pair MAT with a smartphone-based innovation called Addiction CHESS (A-CHESS). A large (*n* = 349) randomized controlled trial (RCT) previously found that A-CHESS decreased risky drinking days and enhanced long-term abstinence among alcohol-dependent people leaving residential treatment, one third of whom reported illicit opioid use [33]. Related field tests in the Veterans Administration and drug courts and among pregnant women in Appalachia [34] also found a positive impact on alcohol and opioid abuse of providing smartphones with A-CHESS. In this trial, we assess the potential of A-CHESS to improve long-term outcomes of MAT among OUD patients. Furthermore, the study seeks to understand—through analyses of mediators and moderators and exploratory analyses—the ways in which A-CHESS works and does not work, for whom, and under what circumstances. Our research team has also developed and pilot-tested systems for improving engagement in care for patients with HIV and improving testing and links to care for patients with HCV. For the present study, these innovations related to HIV and HCV have been incorporated into A-CHESS, allowing us to evaluate whether A-CHESS can also improve screening and treatment outcomes for these conditions.

## Methods/design

### Study design, hypotheses, and outcomes

**Fig. 1:**
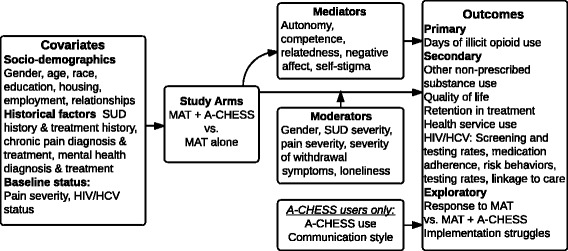
Logic and outcomes of the study

The study, a RCT, will assign 440 opioid users from three addiction treatment centers to receive either MAT + A-CHESS or MAT alone. Patients will be followed for 24 months. The primary hypothesis is that participants assigned to MAT + A-CHESS will have, compared with a control group, a lower percentage of days using illicit opioids. Secondary hypotheses are that those assigned to MAT + A-CHESS will have, compared to the control group, less use of other nonprescribed substances, higher quality of life, greater retention in treatment, and lower health service use. Secondary hypotheses related to HIV/HCV are that those assigned to MAT + A-CHESS will have higher screening and testing rates, greater medication adherence, fewer risk behaviors, and better linkage to care (i.e., referrals that result in in-person visits with providers). We also hypothesize that autonomy (or intrinsic motivation), competence, and relatedness [35] will mediate the effect of MAT + A-CHESS, along with negative affect and self-stigma. We will also determine the person-level factors that moderate the impact of MAT + A-CHESS versus MAT alone (e.g., gender, SUD severity, pain severity, severity of withdrawal symptoms, and loneliness). For patients receiving MAT + A-CHESS, we will examine whether patterns of using A-CHESS and communication style within peer discussion forums are predictors of study outcomes [36]. Figure 1 shows the logic and outcomes for the project. We will use quantitative and qualitative analyses to examine long-term impact, with survey data collected every 4 months during the 24-month period.

### Interventions

#### Control condition: MAT

Patients in the control condition will receive treatment as usual including MAT. Treatment could consist of a recovery plan, medication, and regularly scheduled behavioral interventions such as monthly group counseling sessions, sessions with a substance abuse counselor, and Narcotics Anonymous/Alcoholics Anonymous (NA/AA) meetings. Medication may include methadone, injectable naltrexone, or buprenorphine. The sequence and duration of medication and behavioral interventions will vary by patient and site. Discussions with sites revealed that we should not try to control variations because they are tailored to individual patients. We will document which medications are used and will include as covariates when and how medications change between the 4-month surveys.

#### Experimental condition: MAT + A-CHESS

**Fig. 2:**
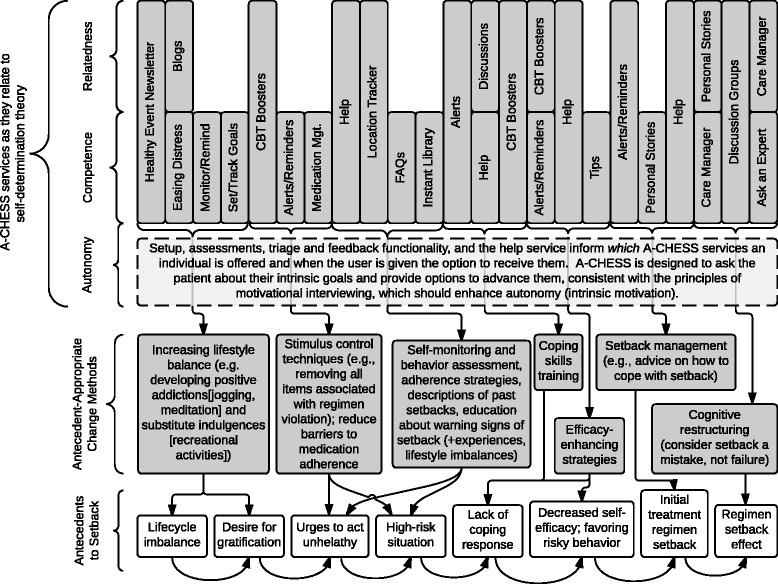
Overview of patient-facing A-CHESS services

**Fig. 3:**
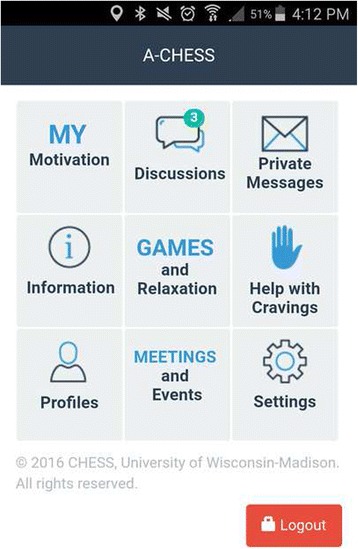
A-CHESS user interface

Patients in the experimental condition will receive a smartphone with A-CHESS for 16 months along with MAT as described above. A-CHESS is designed to improve recovery from addiction. A-CHESS is based on self-determination theory (SDT), which holds that meeting three needs—for autonomy, competence, and relatedness—improves a person’s adaptive functioning [35, 37]. Figure 2 shows how A-CHESS services relate to the constructs of SDT and to the determinants and antecedents of relapse identified by Marlatt [16, 38, 39]. A-CHESS services provide antecedent-appropriate intervention(s) that boost autonomy (intrinsic motivation) by selecting from multiple services those most likely to be most personally meaningful to the patient; offer information, monitoring, and tools to increase competence; and/or increase relatedness. For example, the lower part of Fig. 2 shows antecedents of relapse, one of which is lifestyle imbalance (lower left of figure) and Marlatt’s suggestion that developing substitute indulgences helps (second level-left). The left upper section shows how A-CHESS helps. Another example: A-CHESS monitoring tools include a weekly check-in and GPS-based tracking to identify when lifestyle imbalance may place a patient at risk of using drugs or engaging in unsafe sex. As one healthy alternative, the A-CHESS healthy events calendar may suggest one of the patient’s healthy pleasures, such as going for a walk, and offer a map. We anticipate that this just-in-time approach may be important to help maintain abstinence. Figure 3 shows the A-CHESS user interface. Key A-CHESS services are described below.

Help. When a patient presses *Help*, the system shows a list of the patient’s preapproved supporters and their phone numbers so the patient can easily call for help. The patient can also be linked to positive and potentially distracting activities such as selected games [40] and audio/video-based relaxation recordings [41].

Cognitive behavioral therapy (CBT) boosters offer brief, easy-to-remember reviews of CBT skills that patients learned during treatment to prepare them for future challenges—e.g., how to handle urges and anticipating, avoiding, and mitigating the effects of high-risk people, places, and things related to past drug use.

Monitoring functionality includes the location tracker (described below), self-assessment tools, and a record of A-CHESS use. One self-assessment tool is the Brief Addiction Monitor (BAM) [42], which we implement as a weekly survey. After completing the BAM, participants receive tailored feedback that acknowledges their use of protective behaviors over the past week and provides recommendations for addressing risky behaviors, including links to appropriate A-CHESS content. Participants reporting opioid or other drug use will be encouraged to seek appropriate help.

The location tracker uses GPS to monitor patient movement. If a patient approaches a location that they previously defined as high risk, A-CHESS will initiate a patient-defined recovery process. This might start with a beep, then a vibration, and then a list of preapproved contacts and options for distraction or mindfulness. The GPS service is also often used to find a 12-step meeting. Patients may turn off the location tracker if they perceive certain services to be too burdensome or invasive.

Triage and feedback functionality is designed to derail the relapse process, giving the patient just-in-time, tailored coping support. A-CHESS will be customized at the start by each patient to set options that will be triggered in a moment of need. For example, if a patient reports a craving triggered by environmental cues, such as seeing someone else use, A-CHESS might remind the patient of relaxation exercises, connect them to online peer support and the healthy events calendar, and/or notify a counselor, who may initiate contact via text or private message. Participants whose patterns of using A-CHESS demonstrate they are likely to stop using the system will receive automated messages and tailored messages from coaches to encourage them to reengage.

The counselor dashboard [43], developed by addiction physicians and psychologists, harvests clinically relevant data from A-CHESS and presents it to counselors to help them quickly: (1) identify patients who may be at high risk for relapse and/or benefit from clinical intervention, (2) see a detailed analysis of a patient’s recent history, e.g., trends in individual BAM items, A-CHESS use, and relapse data, and (3) intervene with patients (e.g., through texting in A-CHESS). When a counselor logs into the counselor dashboard, he sees ‘red pins’ generated when A-CHESS (using counselor-determined priorities) detects that a patient may be at high risk. The counselor can adjust the cutoffs for red pins so the ones he sees are most useful.

HIV/HCV services. A-CHESS will integrate components of our team’s existing computerized risk reduction systems that collect data on patients’ HIV/HCV risk behaviors and deliver behavior change interventions tailored to the patient’s self-reported readiness for change. At enrollment, participants in both study conditions will be asked if they have been screened for HIV and HCV. Patients who test negative or decline testing at baseline will be sent reminders from A-CHESS about future testing at a frequency based on reported risk behaviors. Patients found to be HIV or HCV-positive will be provided with targeted multimedia health education content, access to online resources, and location-specific links to clinical care and case management.

Coach-monitored discussion groups [44] foster the exchange of emotional, informational, and instrumental support among patients. Discussions are monitored daily by an A-CHESS coach to encourage appropriate use. Coaches are not counselors, but are members of the research team trained on A-CHESS, risk identification, referral, and technology-based patient engagement. They are skilled in constructive interaction and persistence and are willing to work unusual hours. The coaches encourage individuals to follow up with their health care providers/prescribers regarding medication-assisted treatment questions. We found that a coach increases and sustains use of A-CHESS [45]. Every week, a coach reviews use data. Based on what the coach sees, they write messages to the participants. For example, (1) to a patient active on A-CHESS, “Hi kfields05, Just wanted to say hello and see how things are going. Looks like you are doing a great job of recovery and tracking, which is wonderful. Let me know if there is anything I can help with. Take care and keep it up! Coach Lola.” and (2) to a patient who is not logging in: “Hi Teresa H, Just checking in to see how you are coming with your recovery goals. You have not logged in for a while so I figured I would say hi. Take care and let me know what I can do to help! Coach Lola.”

Possible counselor alerts. A-CHESS sends an email notification to an A-CHESS coach if a patient reports substance use or is over a preset risk threshold on self-monitoring items. The coach may alert a counselor or encourage the patient to seek further support within A-CHESS (e.g., by using discussion groups, games, and relaxation exercises; revisiting their personal recovery motivation; or listening to personal stories from others in recovery) or recommend that the patient seek other professional help.

### Ethics

The study received approval from the Health Sciences Institutional Review Board at the University of Wisconsin-Madison (#2015-1418) and the Western Institutional Review Board (#1163410) in Puyallup, Washington and is registered at ClinicalTrials.gov (NCT02712034). The study complies with the relevant Standard Protocol Items: Recommendations for Interventional Trials (SPIRIT) Statement and World Health Organization Checklist (see the SPIRIT Checklist and figures in Additional files 1 and 2). The study is funded by the United States Department of Health and Human Services National Institute on Drug Abuse.

### Patient eligibility

Patients will be recruited from outpatient detoxification and treatment programs at three sites, two in Massachusetts and one in Wisconsin. Patients are eligible for the study if they (1) are currently on MAT (methadone, injectable naltrexone, or buprenorphine) for their substance use disorder (SUD), (2) are aged 18 years or older, (3) meet criteria for having an OUD of at least moderate severity (4 or 5 *Diagnostic and Statistical Manual of Mental Disorders, fifth edition* (DSM-V) criteria), (4) have no acute medical problem requiring immediate inpatient treatment, (5) have no history of psychotic disorders, though patients with other comorbid psychopathology (mood disorders, anxiety, other substance use disorders) will be eligible, (6) are willing to participate in a randomized clinical trial, (7) provide the name, verified phone number, and address of at least two contacts willing to help locate the patient, if necessary, during follow-up, (8) are able to read and write in English, (9) are not pregnant, (10) are willing to share health-related data with primary care clinicians, and (11) are, at study intake, abstinent from opioids for at least 1 week and no longer than 2 months, except for medications used to treat the disorder.

### Recruitment

**Fig. 4:**
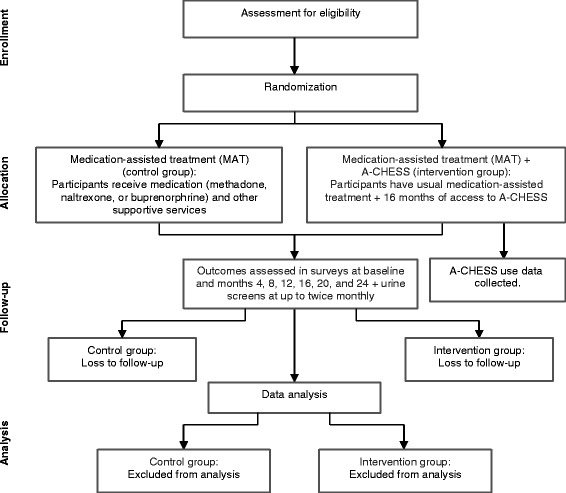
Participant flow

Potential subjects will be identified by a staff person at each of the three sites and asked if they are interested in learning about a study for which they may be eligible. If they answer yes, the University of Wisconsin (UW) or site coordinator will provide a detailed overview of the study, including patient responsibilities and how patient confidentiality will be protected. Interested patients will then provide informed consent, complete a baseline survey, be randomized to receive MAT + A-CHESS or MAT, and, if applicable, be trained on A-CHESS. Figure 4 shows the flow of participants through the trial.

### Randomization

The project director will use a computer-generated allocation sequence to randomize participants in a 1:1 ratio to MAT + A-CHESS or MAT alone, stratifying on gender and site and balancing on age, level of care (intensive outpatient treatment, day treatment, or weekly or monthly counseling), and whether patients had prior SUD treatment. The project director will inform the site coordinator of the group assignment by email; in the email, the participant will be identified only by study ID (the code used to make the identity of participants unknown). The site coordinator will initiate patients into the study condition and, if the patient is assigned to MAT + A-CHESS, provide training.

### Smartphone distribution

Patients randomized to MAT + A-CHESS who do not already have an Android smartphone will be given one loaded with A-CHESS, along with a data plan that includes unlimited data, text, and voice for 16 months. Patients who already have an Android smartphone will have A-CHESS installed on their phone. We will provide up to one replacement phone to patients who report their phone lost, stolen, or broken. If patients lose the second phone, we will offer to load A-CHESS onto an appropriate replacement smartphone (e.g., Samsung S5) that they obtain. We have included in the budget a 20% allowance for replacement phones, which has proven sufficient in prior CHESS research, including trials with populations of addicted patients [33, 46].

### Training to use A-CHESS

The UW or site coordinator will train patients to use the A-CHESS app and customize it − e.g., by sources of support (such as family), contacts who detract from recovery (such as friends who use illegal drugs) and support recovery, and so on. A-CHESS will be updated monthly with activities for the healthy event calendar; changes, if any, to therapeutic goals and the recovery plan (e.g., self-help groups, medication) and in home, work, or educational responsibilities; and high-risk locations to avoid. Patients must demonstrate that they can use A-CHESS (e.g., make one post to the discussion group) before they leave the training session with the phone.

### Quantitative data collection

All subjects will complete follow-up surveys over the phone with the UW study coordinator at months 4, 8, 12, 16, 20, and 24. Data collected will relate to the variables shown in Fig. 1. Each phone survey is expected to take 20 to 30 min. Surveys will be identified by study ID, not participant name. The form linking study IDs and names will be kept in REDCap [47]. In addition, urine screens, which are done routinely as part of MAT, will be conducted at each study site and recorded for all subjects at baseline and up to twice a month thereafter. Results will be used to validate self-reported information. Inconsistent results between urine drug tests and self-report results will not affect patients’ ability to continue in the study.

### Qualitative data collection

We will administer in-depth interviews with patients to shed light on their perspectives as well as on what promotes and hinders implementing and sustaining MAT + A-CHESS. We will explore patient perceptions of the effects of integrating MAT + A-CHESS, the most and least useful services in A-CHESS, gender-specific effects, and how patients feel about various A-CHESS services over time. A second set of interviews will examine provider perceptions of benefits of and barriers to integrating and sustaining MAT + A-CHESS over time. A third set of interviews will examine fine-tuning MAT + A-CHESS; communication between the research team, patients, and providers; and concerns from providers and patients. These data will help to refine methods for developing mHealth systems generally.

UW research staff will also conduct a longitudinal case study of five female and five male patients to explicate MAT + A-CHESS effects, considering patients’ medical and addiction treatment history, family history, personal and gender-specific preferences, and environmental factors. Case studies, though underused in health care [48], are a good way to understand how innovations work in real life [49]. They provide insight into patterns that might be overlooked in RCTs because they reveal the complexities of systems in which innovations are introduced. By following these ten individuals over time, we will explore how women and men integrate new technologies into their lives, circumstances that favor or complicate the process, and barriers to sustainability. Data collection from interviews and focus groups will ensure the comprehensiveness of findings and strengthen validity [50].

### Measures

All scales have good psychometric properties with similar populations. Listed below are the factors to be measured and instruments to be used, along with references to validation studies for the instruments.

Intake and baseline. Treatment center staff will document patient eligibility. Then patients will complete the baseline survey. They will report 12 demographic items (gender, age, race, education, etc.) and five items related to their opiate use history (age of first use, age of regular use, whether opioid use began with a physician-prescribed opioid, number of past quit attempts, and date of last use). Patients will also respond to items related to chronic pain diagnosis and treatment, current and past comorbid psychiatric diagnoses and treatment, pain severity (using the Numeric Rating Scale (NRS-11)) [51], and HIV/HCV status.

Dependent variable. Self-reported illicit opioid use days will be analyzed in 30-day periods. At baseline, a Timeline Followback (TLFB) [52] for the 30 days before admission will be obtained (with opioid use separated from other drug use) and a urine drug screen collected (CTN-approved drug use outcome measures). For follow-up assessments, the TLFB for the previous 120 days will be obtained. The TLFB has been successfully used to obtain drug use data for extended periods of time and with polydrug-using patients [53].

At months 4, 8, 12, 16, 20, and 24. During phone surveys after baseline data collection, patients will complete a 120-day TLFB [54] to document their illicit opioid and other nonprescribed drug and alcohol use as well as health service use during the past 4 months. Health service use will be collected for the categories listed under “Health service use and cost” below. Patients will also complete measures of: relapse risk (Brief Addiction Monitor [55], nine items); pain severity (NRS scale [56], one item); HIV/HCV screening and link-to-care status (testing status and if tested, result, and if positive, whether the patient saw a medical provider); risk behaviors (HRBS [57], five items); status of current housing (two items) and employment (three items on type of employment, hours worked); quality of life (The Satisfaction with Life Scale [58], eight items); rating scale for withdrawal (three items); self-reported medication adherence (Morisky Medication Adherence Scale: MMAS-4 [59], four items); and loneliness (three items). We will collect number of phones lost, stolen, or broken (research records). For retention in treatment, we will take the proportion of appointments attended from clinic records. We will also determine if participants are engaging in other forms of treatment outside of the treatment facility, such as seeing a therapist, working with a sponsor, or attending NA/AA meetings.

Mediators. Self-determination theory (SDT) constructs will be assessed as follows: autonomy, Treatment Self-Regulation Questionnaire [60] (six items); competence, revised Drug-Taking Confidence Questionnaire [61] (eight items); and relatedness, our own bonding scale (five items). Negative affect will be assessed by Positive and Negative Affect: PANAS [62, 63] (20 items), and self-stigma by the self-devaluation subscale of the self-stigma in substance abuse scale [64] (seven items).

Moderators: we will focus our evaluation on gender but also collect data on other potential moderators (SUD severity as determined by the treatment site at intake using DSM-V criteria, pain as determined by the NRS-11, withdrawal, and loneliness).

A-CHESS use. A-CHESS use will be collected in time-stamped log files and includes when a patient accessed A-CHESS, service(s) selected, duration of service use, pages viewed, messages posted versus received, and communication style and content of messages. Content will be subject to computer-automated content analysis to identify communication styles that may predict study outcomes. Cumulative use (number of pages viewed and days used) significantly predicted risky drinking days in the randomized trial of A-CHESS with alcohol-dependent patients [33]. We will also collect data on sources of other SUD-related information and support.

Health service use and cost. Our cost analysis is motivated by the potential that A-CHESS has shown to reduce the use of costly health services associated with relapse. (In a field test with US military veterans, A-CHESS users had substantially decreased rehospitalizations related to relapse.) Our approach to measuring and analyzing health use data is adapted from McCollister and French’s 2003 analysis of the economic benefit of addiction interventions [65]. We will use the following categories of health service use: emergency room visits, hospital detox (day), and short-term residential treatment (day). We will also track costs for all other hospital visits and stays (in addition to emergency room visits and hospital detox); urgent care visits for any reason (to which we will apply cost estimates derived from a national survey of urgent care clinics [66]; individual psychotherapy or psychiatric care; and self-reported outpatient addiction treatment services after relapse (using the categories of outpatient addiction care outlined by McCollister and French [64], to which we will apply cost estimates provided in the 2008 national survey of 110 substance abuse treatment programs by French et al., adjusted for inflation [67]). We will also collect self-reported use of other health services (e.g., dental care, primary care, chiropractor), following the approach by Bell et al., to assess the cost-effectiveness of supervised versus unsupervised buprenorphine-naloxone administration [68]. We will derive cost estimates for various types of health care use (e.g., emergency room visits, hospitalizations, primary care visits, etc.) using data from the American Hospital Association and the American Medical Association.

### Timeline

**Fig. 5:**
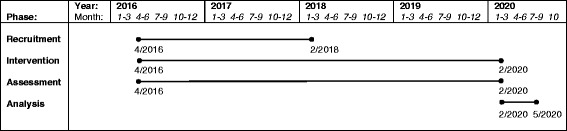
Study timeline

Recruitment began in April 2016 and will end in February 2018. The intervention period will end in February 2020. Figure 5 shows the study timeline.

### Data analysis

#### Power and sample size justification

Primary analysis. Our study will be powered to detect a difference between MAT + A-CHESS and MAT in percentage of days using illicit opioids across the 24-month intervention period. Based on data from the two sites from which most study participants will be recruited, we assume 35% attrition over the course of the study, providing an *N* of 286. Using the power program by Hedeker et al. [69] and assuming up to cubic trends in the data, given expected attrition, recruitment of 440 patients will provide 80% power to uncover a standardized difference of .35 across the 24 months. From prior data [70, 71], this would be a difference of approximately three opioid use days/month depending on the observed standard deviation.

Secondary analyses. Power for examining intervention effects for the secondary outcomes would be similar as for the percentage of opioid use days, though the difference implied by the standardized difference of .35 will depend on the actual standard deviation of each measure. For secondary outcomes related to HIV and HCV, the sample size also provides 80% power to find a two-tailed difference in proportion screened for HIV/HCV of 16% [72] (conservatively assuming that screening for one group nears 50%, the point requiring the largest difference to achieve a specified power).

Mediation and moderation analyses. Power for detecting specific parameter changes in the structural model will be estimated using a procedure proposed by Satorra and Saris [73] that approximates the noncentral chi-square distribution. A total *N* of approximately 220 patients would provide adequate power (>.80) to detect a group difference in a parameter by 0.4 standard deviations (a moderate effect). Since our primary analysis projects a final *N* of 286, we are confident our secondary process analysis will have adequate power.

Missing data. In previous addiction work, we completed 85% of surveys at 4, 8, and 12 months. We anticipate greater reductions at months 16, 20, and 24, reaching about 65% by month 24. In previous studies, we have kept missing data on core items in a survey to about 2% and expect a comparable rate in this study. In addiction treatment, data are not likely to be missing at random (i.e., the probability that data are missing is related to what the data would have been had the data been observed). For example, some patients may not want to disclose opioid use in surveys. Because this may lead to biased parameter estimates for our models, we will identify missing data patterns and use pattern-mixture modeling to test the sensitivity of our longitudinal intervention analysis to missing data assumptions [74–77] and conduct other sensitivity analyses after imputing missing data with a range of clinically plausible values based on explicit assumptions for the missing data (e.g., best-case, worst-case; with and without multiple imputation) [78–80].

Intention-to-treat and subject noncompliance. Standard intention-to-treat (ITT) estimates the average treatment effect by comparing outcomes based on assignment to treatment, but ignoring use of the treatment. Because ITT estimates do not represent treatment efficacy under noncompliance (e.g., a patient is randomized to A-CHESS but does not use the system), we will address noncompliance by also estimating treatment effects only for compliers using as-treated, per-protocol [81], and CACE (Complier Average Causal Effect) [82].

Dropout rates. In our A-CHESS RCT with alcohol-dependent patients [33], 88 patients were using opioids as well as alcohol; 261 were not using opioids. We compared the post-test survey response rate of opioid-using patients to the response rate of patients who did not use opioids. The non-opioid-using patients’ response rates were 94.3% at 4 months; 90.6% at 8 months, and 86.7% at 12 months. The opioid-using patients’ response rates were 91.2%, 86%, and 79.1%. Response rates declined in a relatively linear fashion in both groups, with reductions of about 5 percentage points in each period. We assumed a 65% response rate at 24 months by continuing the drop off rate for each of the three periods from 79% to 74% to 69% to 64%. Hence, we believe it is likely that by the end of the study we will still be able to reach 65% of patients originally enrolled.

Mediation analysis. Mediator variables will all be collected in the first two visits (at the 4- and 8-month visits) while the outcome (illicit drug use days) will be collected at months 12, 16, 20, and 24. Because mediator-outcome relations might reflect the effects of drug use while the mediator is being assessed (e.g., drug use might suppress ratings of competence), drug use that occurs during the mediator assessment period will be covaried out of models to examine and control its influence. Moreover, to assess the nonorthogonality of the mediators (which seems likely with the self-determination variables), we will use multiple mediator analyses based on a Bayesian approach illustrated in Yuan and MacKinnon [83]. This Bayesian estimation of the meditational models can be implemented through Markov Chain Monte Carlo (MCMC) techniques. Unlike more traditional estimation methods, such as maximum likelihood or least squares methods, for example, MCMC methods rely on sampling techniques to estimate model parameters and resulting mediation effects (i.e., iterative sampling from the parameter distributions is used to estimate confidence intervals to identify significant effects). An appealing feature of the method is its relative ease of implementation, particularly for complex statistical models. Similar to Yuan and MacKinnon, we will implement MCMC using WinBUGS 1.4 [84]. The multiple mediator models will be conducted with only those mediators shown to be significant in univariate models. See Bolt et al. [85] for our previous application of this analytic approach.

Qualitative analysis. Content analysis [86] of interview transcripts will describe the role that MAT + A-CHESS plays in sustaining opioid recovery and reducing HIV/HCV risk; identify potential improvements in MAT and A-CHESS; and supplement the quantitative analysis. UW research staff will: (1) construct a coding scheme [87] by combining categories flowing from the research questions, categories used in previous studies, and a preliminary examination of the data. Ideas will be our unit of analysis (rather than words or paragraphs) to capture references to a concept as well as direct statements about it, (2) test the coding scheme on a sample of data. Three coders will independently code the data in NVivo. We will calculate intercoder reliability and develop a set of coding instructions to insure reliability of at least .80, (3) code the full dataset and create a conceptual model to help explain the mechanisms by which, and conditions under which, the interventions affect opioid use and HIV/HCV screening. These conclusions will help us understand the benefits of and modifications needed for MAT + A-CHESS (and MAT alone) to sustain recovery over the long term.

## Discussion

This study is the first to our knowledge to test whether MAT for OUDs, when combined with a smartphone-based relapse prevention system, can significantly improve long-term recovery from opioid dependence when compared with MAT alone. The study will also explore for whom, and under what circumstances, A-CHESS does and does not work, and whether the tested bundle of services can reduce relapse-related health service use.

We believe that the HIV/HCV component of the study adds value to the intervention in two ways: (1) the prevalence of HIV/HCV infection is high among opioid-using populations, yet most addiction treatment centers do not perform routine testing; bundling HIV/HCV services with A-CHESS could improve screening rates in a high-risk population for two serious but highly treatable conditions, (2) screening for HIV/HCV is consistent with the project’s overall goal of improving access to comprehensive health services for opioid-dependent patients, rather than focusing narrowly on promoting abstinence from opioids. We recognize that, despite the availability of evidence-based interventions, many patients who have injected opioids will relapse. The bundled intervention seeks to meet a public health goal of reducing the number of people who are infected with HIV or HCV but are unaware of being infected and, therefore, continue to place others at risk.

### Public health impact

mHealth systems can attend to patients nearly as constantly as addiction does. At the end of this project, we will understand whether bundling MAT with an mHealth relapse prevention system can improve long-term recovery from opioid dependence. Just as important, we will better understand factors that will improve the design and delivery of treatment. This new knowledge could have wide and lasting benefits for patients who suffer from SUDs and other chronic conditions and for the health systems designed to help them.

### Trial status

The trial has received ethical approval and recruited 23 participants to date (8 June 2016). We anticipate ending recruitment in February 2018.

## Additional files

Additional file 1: Completed SPIRIT Checklist, the addendum to which contains the completed WHO Checklist. (DOC 148 kb)
Additional file 2: Completed SPIRIT figure. (PDF 103 kb)

## Abbreviations

A-CHESS: Addiction CHESS (Comprehensive Health Enhancement Support System)
BAM: Brief Addiction Monitor
DSM-V: *Diagnostic and statistical manual of mental disorders*, *fifth edition*
HCV: Hepatitis C virus
HIV: Human immunodeficiency virus
MAT: Medication-assisted treatment
NA/AA: Narcotics Anonymous, Alcoholics Anonymous
OUD: Opioid use disorder
SUD: Substance use disorder
