# Supplementary material for: The effect of bundling medication-assisted treatment for opioid addiction with mHealth: study protocol for a randomized clinical trial
Source: Trials. 2016 Dec 12;17:592. doi: 10.1186/s13063-016-1726-1 (PMC5153683; doi:10.1186/s13063-016-1726-1)
Supplement: Additional file 2: — Completed SPIRIT figure. (PDF 103 kb) [file 13063_2016_1726_MOESM2_ESM.pdf]

ADDITIONAL FILE 2: SPIRIT figure

	STUDY PERIOD									CLOSE-OUT
	**Enrollment**	**Allocation**	**Post-Allocation Month**							
**TIMEPOINT**	**4/2016 – 2/2018**	**4/2016 – 2/2018**	**0**	**4**	**8**	**12**	**16**	**20**	**24**	**2/2020-5/2020**
**ENROLLMENT:**										
Eligibility screen	X									
Informed consent	X									
Other procedures			For MAT+A-CHESS group: A-CHESS training							
Allocation		X								
**INTERVENTIONS:**										
MAT + A-CHESS			◆	—	—	—	◆			Data cleaning, analysis, and publica-tions
MAT Alone			◆	—	—	—	◆			
Other study groups	N/A									
**ASSESSMENTS:**										
Baseline variables:										
Gender, age, race, education, SUD history & treatment history, chronic pain diagnosis & treatment history, mental health diagnosis & treatment history			X							
Housing, employment, relationships, pain severity, HIV/HCV status			X	X	X	X	X	X	X	

Continued on next page

**SPIRIT figure** *continued*										
	**STUDY PERIOD**									**CLOSE-OUT**
	**Enrollment**	**Allocation**	**Post-Allocation Month**							
**TIMEPOINT**	**4/2016-2/2018**	**4/2016-2/2018**	**0**	**4**	**8**	**12**	**16**	**18**	**24**	**2/2020-5/2020**
**ASSESSMENTS** *continued:*										Data cleaning, analysis, and publications
Outcome variables:										
Primary: Days of illicit opioid use. *Secondary:* Other non-prescribed substance use; quality of life; retention in treatment; health service use; HIV/HCV: Screening and testing rates, medication adherence, risk behaviors, testing rates, linkage to care. *Exploratory:* Response to MAT vs. MAT+A-CHESS, implementation struggles.			X	X	X	X	X	X	X	
Mediators: Autonomy, competence, relatedness, negative affect, self-stigma			X	X	X	X	X	X	X	
Moderator, assessed only at baseline: Gender			X							
Other moderators: SUD severity, pain severity, severity of withdrawal symptoms, loneliness			X	X	X	X	X	X	X	
A-CHESS users only: A-CHESS use, communication style			A-CHESS use and participant communication style are continuously monitored electronically during the study period.							
